# Phase-vanishing halolactonization of neat substrates

**DOI:** 10.3762/bjoc.4.29

**Published:** 2008-08-11

**Authors:** Nicole Windmon, Veljko Dragojlovic

**Affiliations:** 1 Department of Chemistry, Florida Atlantic University, 777 Glades Road, Boca Raton, Florida 33431, USA; 2 Wilkes Honors College of Florida Atlantic University, 5353 Parkside Drive, Jupiter, Florida, 33458, USA.

**Keywords:** bromine, halocyclization, halolactonization, iodine monochloride, phase-vanishing reactions

## Abstract

Phase-vanishing reactions are triphasic reactions which involve a reagent, a liquid perfluoroalkane as a phase screen and a substrate. The perfluoroalkane does not dissolve any of the reactants and is used to separate them. Halolactonization of neat substrates under phase-vanishing conditions avoids use of both solvents and basic reaction conditions. Both γ,δ-alkenoic acids as well as the corresponding methyl esters are suitable substrates for phase-vanishing halolactonizations. The reaction works well both on solid and liquid substrates and the products are obtained in good to excellent yields, particularly in the case of rigid bicyclic systems. Bromine (Br_2_) and iodine monochloride (ICl) are suitable electrophiles for bromolactonization and iodolactonization, respectively. Although in some cases iodine gave satisfactory yields of the corresponding iodolactone, it is generally inferior to iodine monochloride.

## Introduction

Phase-vanishing reactions, introduced by Ryu [1], Curran [1–2] and Verkade [3], are triphasic reactions, which involve a reagent, a substrate and a liquid perfluoroalkane. A more recent improvement of phase-vanishing brominations is addition of water as the fourth phase to act as an acid scavenger [4]. The perfluoroalkane does not dissolve the reactants and is used as a phase screen to separate them. In this procedure, the halogen reagent is of a higher density while the substrate is of a lower density than the perfluoroalkane phase screen. As the reagent in the lower layer diffuses through the perfluoroalkane layer, it reaches the top layer and reacts with it. Since the rate of diffusion is relatively low, the reaction proceeds at a moderate rate, instead of a vigorous, often violent, reaction that would occur if the two reactants were mixed without a phase screen. In the course of the reaction, the reagent disappears (“vanishes”) and the product is mechanically separated from perfluoroalkane, which can be reused.

Halolactonization and halocyclizations are important reactions in organic synthesis and structure determination [5–6]. Traditionally, a halolactonization is done in a mixture of aqueous solvent and an organic co-solvent in the presence of a base, such as sodium bicarbonate [7–8]. Reagents commonly used in halolactonization are I_2_/NaHCO_3_ [9], Br_2_ [10], IBr [11]and ICl [12]. We investigated phase-vanishing reaction conditions as a more efficient and an environmentally friendly alternative to traditional halolactonizations. Reactions were done on neat reagents, which avoided use of any solvent other then a phase screen – perfluorohexane (FC-72), simplified the work up and improved the yields.

## Results and Discussion

Bromolactonization worked very well and was easy to monitor as it was a true “phase-vanishing” reaction. The bromine layer disappeared at the end of the reaction and the products were obtained in good to excellent yields. However, sometimes dibromo derivatives, resulting from addition of bromine to a double bond, were by-products. The reaction mechanism of halolactonization in basic aqueous medium is believed to involve formation of a halonium ion followed by attack of an oxygen nucleophile [13].

Reaction times ranged from 20–60 minutes when Br_2_ or ICl were used to several days when I_2_ was used. Reaction rates can be controlled by the amount of FC-72 and the rate of stirring – a larger amount (greater depth) of FC-72 resulted in a slower reaction, while increased rate of stirring resulted in a faster reaction. If a very slow rate is desired, the reaction can be done without any stirring. Very fast stirring is not recommended as it may lead to direct mixing of the top and bottom phases and a violent reaction. When a reaction is done with stirring, it is recommended that a vial be clamped and not just placed on the top of a magnetic stirrer. Sometimes dense, solid products form, which encase the stirring bar. A result is that, if not clamped, the entire vial may begin to rotate and it may flip over. Usually, solubility of the reaction products in FC-72 is negligible and it can be reused. However, one should always check the fluorous phase for presence of the product. Finally, due to a low boiling point of FC-72, a considerable amount of it may evaporate in the course of longer reactions unless the reaction vessel is capped. Capping a vial is not always feasible as the reaction byproduct is a gas. In our hands, on ~5 g scale, there were no problems when reaction was done in a capped 20 mL vial.

**Scheme 1:**
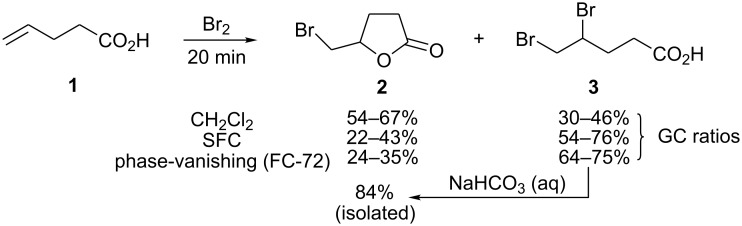
Bromolactonization of 4-pentenoic acid.

In the case of bromination of 4-pentenoic acid (**1**), under phase-vanishing conditions there was competing formation of 4,5-dibromopentenoic acid (**3**). Formation of the dibromo derivative as a minor product in the course of bromolactonization of 4-pentenoic acid has been reported along with the observation that it readily cyclizes to give the corresponding bromolactone [14–15]. For comparison, the reaction was done in dichloromethane and directly on neat reagents under solvent-free conditions (SFC) (***caution: an extremely violent reaction!***). In dichloromethane as a solvent, bromolactone **2** was produced in a slight excess. Under SFC, the reaction was surprisingly clean in that it gave only a mixture of the bromolactone **2** and dibromoacid **3**. The ratios of the two varied between different runs with the dibromide **3** predominating. Outcome of the reaction under phase-vanishing conditions on neat reactants closely resembled an SFC reaction (Scheme 1, see Supporting Information File 1 and Supporting Information File 2 for full experimental data). 4,5-Dibromopentanoic acid was identified based on its mass spectrum and was not isolated. Instead, the crude reaction mixture was treated with aqueous sodium bicarbonate and the resulting bromolactone **2** was isolated. Thus, unlike other substrates in this study, 4-pentenoic acid required basic reaction conditions for successful bromolactonization. As bromolactonization of methyl esters **17** and **20** worked very well (*vide infra*), bromolactonization of methyl 4-pentenoate was attempted. The major product was methyl 4,5-dibromopentanoate (~80%) and the bromolactone was only a minor product (~20%).

**Scheme 2:**
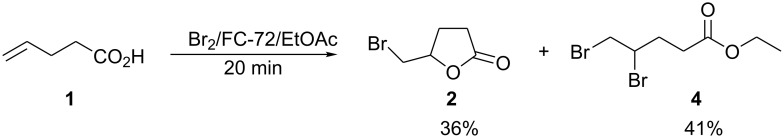
Bromolactonization of 4-pentenoic acid in ethyl acetate.

Interestingly, when the reaction was done in ethyl acetate, or ethyl acetate was used in a work up, the major isolated product was ethyl 4,5-dibromopentanoate (**4**) (Scheme 2). Apparently, 4,5-dibromopentanoic acid underwent hydrogen bromide-catalyzed transesterification with ethyl acetate. In a control experiment, 4-pentenoic acid was transesterified by dissolving it in ethyl acetate and adding a catalytic amount of HBr in acetic acid. The best results, and the best yields of bromolactone, were obtained under phase-vanishing conditions when, upon consumption of bromine, FC-72 was removed and the residue was treated with aqueous NaHCO_3_, followed by extraction with ethyl acetate. It should be pointed out that this was the only bromolactonization where we encountered problems. Bromolactonization of other compounds (**7**, **14**, **17** and **20**) worked well.

**Scheme 3:**
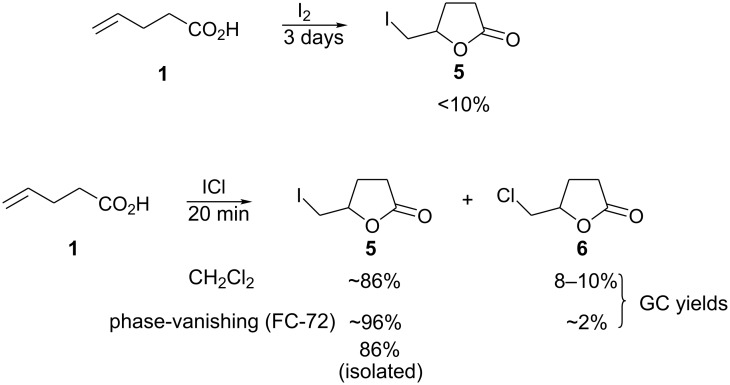
Iodolactonization of 4-pentenoic acid.

While reaction of 4-pentenoic acid with bromine gave the corresponding bromolactone in a high yield, a reaction with iodine did not give good results (Scheme 3). The reaction was slow and the product was apparently unstable. Thus, the corresponding iodolactone **5**, along with a number of unidentified byproducts, was observed in GC-MS analysis of the reaction mixture. Isolation of the reaction product was not attempted. When iodine monochloride was used in place of iodine, the reaction was fast and the corresponding iodolactone **5** was obtained cleanly and in a high yield. The only byproduct (<2% according to GC-MS), which was not isolated and was not characterized, had a mass that corresponded to chlorolactone **6**. When iodolactonization of 4-pentenoic acid was done in dichloromethane, the same byproduct formed in a larger amount (8–10%).

**Scheme 4:**
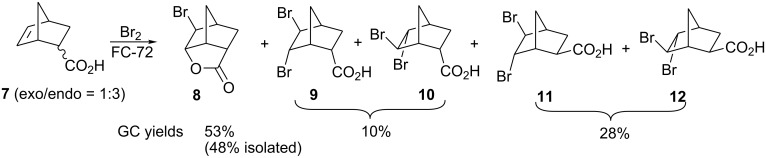
Bromolactonization of 5-norbornene-2-carboxylic acid.

Treatment of 5-norbornene-2-carboxylic acid (**7**) (3:1 mixture of *endo* and *exo* isomers) gave the expected bromolactone **8** along with four byproducts (Scheme 4). GC analysis showed the four byproducts as two pairs of closely spaced peaks (retention times of 8.1 and 8.3 min for one pair and 12.5 and 12.6 min for the other pair). Furthermore, GC-MS analysis indicated that the byproducts were the corresponding dibromo compounds. They were tentatively assigned structures **9**–**12**. It was expected that one pair of the dibromo compounds (**11** and **12**) would form as *exo*-5-norbornene-2-carboxylic acid cannot cyclize and, indeed, a pair of dibromo compounds formed in the amount that approximately corresponded to the amount of the starting *exo*-5-norbornene carboxylic acid. Another pair of dibromo compounds (assumed to have structures **9** and **10**) formed in a combined yield of ~10% (according to GC-MS analysis). Thus, formation of dibromo products decreased compared to 4-pentenoic acid. Interestingly, no dibromo derivatives at all were observed in bromolactonizations of diacid **14** and diesters **17** and **20** (Table 1, entries 3, 6 and 8).

**Table 1:** Phase-vanishing halolactonization.

Entry	Starting Material	Reagent (equiv)	Product (isolated yield, %)	Reaction time

1	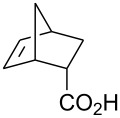 **7** (3:1 *endo*/*exo*)	Br_2_ (1.05)	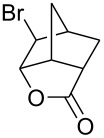 **8** (48%, 64% with respect to *endo*)	1 h
2	**7** (3:1 *endo*/*exo*)	ICl (1.15)	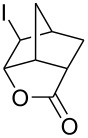 **13** (65%, 87% with respect to *endo*)	1 h
3	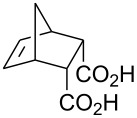 **14**	Br_2_ (1.05)	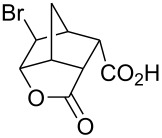 **15** (72%)^a^	1 h
4	**14**	ICl (1.15)	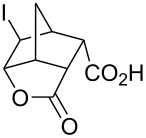 **16** (75%)^a^	1 h
5	**14**	I_2_ (1.20)	**16** (54%)	3 days
6	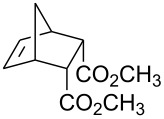 **17**	Br_2_ (1.05)	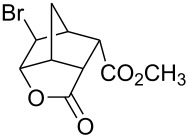 **18** (83%)	1 h
7	**17**	ICl (1.15)	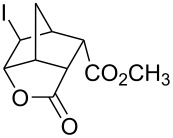 **19** (86%)	1 h
8	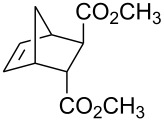 **20**	Br_2_ (1.05)	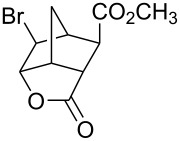 **21** (92%)	30 min
9	**20**	ICl (1.15)	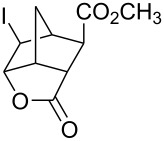 **22** (94%)	1 h

^a^Isolated as the corresponding methyl esters.

Iodine monochloride also worked very well on the same substrates (Table 1, entries 2, 4, 7 and 9). However, the reaction was more difficult to monitor. As commercially available iodine monochloride contains an excess of iodine, one has to use an excess of the reagent and, at the end of the reaction, there was some unreacted iodine left over. Thus, one cannot tell when the reaction is over and reaction times were more difficult to determine. Reaction times provided in the Table 1 are the times when the reaction was definitively completed. The actual reaction times were probably shorter. As in the case of 4-pentenoic acid, iodolactonization of compounds **7**, **14** and **17** was accompanied by formation of a small amount of a byproduct (<2% according to GC-MS), which were not isolated. Mass spectra of the byproducts were consistent with the corresponding chlorolactones. Under the phase vanishing conditions, the amounts of chlorolactones were relatively small. In dichloromethane the amounts increased to as much as 10% (GC-MS). Interestingly, in both bromolactonization and iodolactonization, diester **17** gave cleaner products and better yields compared to the corresponding diacid **14**. *trans*-Diester **20** gave the best yields in both bromo- and iodolactonization reactions. Neither dibromo compounds (in a reaction with bromine) nor chlorolactone (in a reaction with iodine monochloride) were observed and the corresponding crude products gave single peaks when analyzed by GC and were >95% pure by ^1^H NMR.

It has been reported that iodolatonization of the diacid **14** with iodine failed, while iodolactonization of the corresponding disodium salt gave a mixture of γ- and δ-lactones [16]. However, no experimental details were given. Under phase-vanishing conditions, treatment of **14** with iodine gave the corresponding γ-lactone **16** in a moderate yield. The yield was inferior compared to iodolactonization with iodine monochloride. On the other hand, iodine is a safer reagent and one may consider using it under certain circumstances (e.g., a large scale reaction, or need for an inexpensive reagent rather than a high yield).

Halolactonization reaction worked well both on neat liquid and solid substrates. Although the diesters **17** and **20** are solids, their reactions proceeded through formation of melts [17] and gave the corresponding halolactones in high yields (Table 1, entries 6–9). However, stirring was necessary as the respective products were also solids and sometimes they solidified at the interface of FC-72 and ester layers, preventing further reaction. Good stirring prevented formation of a solid layer. Alternatively, the solid layer was occasionally stirred with a small glass rod (a sealed capillary melting point tube) to break the clumps. Solid acid **14** reacted without an apparent formation of a melt and that may explain why the yields were somewhat lower compared to the corresponding diester **17**.

## Conclusion

In conclusion, a halolactonization of both solid and liquid neat reactants under phase-vanishing conditions is a simple and efficient approach to various halolactones. While a number of methods to prepare those compounds in high yields have been reported, the methodology described avoids use of any solvent other than the phase screen, the reaction was done under neutral reaction conditions, and work-up consisted of mechanical separation of the product from the phase screen. An exception was bromolactonizaiton of 4-pentenoic acid, which required a basic workup. The products were isolated in high yields and require little or no purification. The best electrophiles were Br_2_ and ICl while I_2_ gave somewhat inferior results. The procedure worked particularly well on methyl esters of the γ,δ-alkenoic acids.

## Experimental

All of the commercially available reagents (bromine, iodine, iodine monochloride, FC-72, 4-pentenoic acid, dicyclopentadiene, acrylic acid, maleic anhydride, dimethyl maleate and dimethyl fumarate) were used as supplied without further purification. Compounds **7**, **17** and **20** were prepared in Diels-Alder reactions between cyclopentadiene and the corresponding dienophiles. Diacid **14** was prepared in a reaction between cyclopentadiene and maleic anhydride followed by aqueous hydrolysis. Those compounds gave satisfactory GC-MS and ^1^H NMR spectra.

Reactions were done in glass vials of various sizes (4–20 mL). Halogen (2.2–30.0 mmol) was added first, followed by of FC-72 (0.5–5 mL) and finally 2.0–25.0 mmol of an alkene. After stirring for 20 min – 3 days, the product was isolated by separating it from the fluorous phase either by filtration (solid products) or by removing the fluorous phase with a pipette.

## Supporting Information

File 1 Experimental part

File 2 GC-MS and NMR spectra
