# Supplementary material for: Phase-vanishing halolactonization of neat substrates
Source: Beilstein J Org Chem. 2008 Aug 11;4:29. doi: 10.3762/bjoc.4.29 (PMC2533434; doi:10.3762/bjoc.4.29)
Supplement: File 1 — Experimental part [file Beilstein_J_Org_Chem-04-29-s001.doc]

**Supporting Information**

**Phase-vanishing halolactonization of neat substrates**

Nicole Windmon^1^ and Veljko Dragojlovic^2,*^

^1^Department of Chemistry, Florida Atlantic University, 777 Glades Road, Boca Raton, Florida 33431, USA.

^2^Wilkes Honors College of Florida Atlantic University, 5353 Parkside Drive, Jupiter, Florida, 33458, USA.

Veljko Dragojlovic – [vdragojl@fau.edu](mailto:vdragojl@fau.edu)

*corresponding author

1. General experimental methods.

Separations of the reaction products were done either by column chromatography or by preparative radial thin layer chromatography (Harrison Chromatotron). GC-MS analyses were performed by means of Agilent 6890N Gas Chromatograph equipped with HP-5MS 30 m x 0.25 mm column and Agilent 5973N MSD. ^1^H NMR spectra were recorded on a 400 MHz spectrometer in CDCl_3_ solutions. All of the isolated products were known compounds and gave satisfactory GC-MS and ^1^H NMR data. ^1^H NMR spectra of the compounds (**2** [1], **5** [2], **8** [3], **13** [4], **19** [5] and **22** [6] have been reported in the literature. Compounds **4** [7]**, 18** and **21** [8] have been reported. However, it appears that their ^1^H NMR spectra have not been reported so far.

1. Experimental procedures

**2**

**5-(Bromomethyl)-dihydrofuran-2(3*H*)-one** (**2**) – phase-vanishing reaction. A stirring bar, 0.20 mL of bromine, 3.0 mL of FC-72 and 0.40 mL of 4-pentenoic acid were placed in a 4 mL vial. The solution was stirred for 20 min. (until the color of bromine disappeared). The remaining phase screen was removed on a rotary evaporator, or simply allowed to evaporate in a fumehood. The oily residue was treated with a saturated solution of sodium bicarbonate (4 mL) and extracted with ethyl acetate (2 x 3 mL). Ethyl acetate extracts were combined, dried (anh. MgSO_4_) and evaporated. Chromatography (Harrison Chromatotron, eluting with 3:1 hexanes/ethyl acetate) gave 0.60 g (84%) of 5-(bromomethyl)-dihydrofuran-2(3*H*)-one (**2**).

**5-(Bromomethyl)-dihydrofuran-2(3*H*)-one** (**2**) – neat reactants. Bromine (0.20 mL), 1.0 mL of FC-72 and 0.40 mL of 4-pentenoic acid were placed in a 125 mL separatory funnel. Funnel was tilted until bromine came into contact with 4-pentenoic acid (***caution: very vigorous reaction, work in a fumehood!***). The funnel was gently shaken until all of the bromine was consumed. The product was dissolved in dichloromethane, analyzed by GC-MS (page S3 in Supporting Information File 2) and isolated as described above.

**5-(Bromomethyl)-dihydrofuran-2(3*H*)-one** (**2**) –reaction in dichloromethane. A stirring bar and a solution of 0.40 mL 4-pentenoic acid in a 4 mL dichloromethane were placed in a 25 mL round bottom flask. A 10% solution of bromine in dichloromethane was added until a pale yellow color of bromine persisted. The reaction products were analyzed by GC-MS and isolated as described above.

**4**

**Ethyl 4,5-dibromopentanoate** (**4**). A stirring bar, 0.20 mL of bromine, 3.0 mL of FC-72 and a solution of 0.40 mL of 4-pentenoic acid in 4 mL of ethyl acetate were placed in a 20 mL vial. The solution was stirred for 1 h (until the color of bromine disappeared). The layers were separated and the ethyl acetate layer was treated twice with a saturated solution of sodium bicarbonate (3 mL each time). The aqueous layers were combined and extracted with ethyl acetate (3 x 3 mL). Ethyl acetate extracts were combined, dried (anh. MgSO_4_) and evaporated. Chromatography (Harrison Chromatotron, eluting with 10:1 hexanes/ethyl acetate) gave 0.26 g (36%) of 5-(bromomethyl)-dihydrofuran-2(3*H*)-one (**2**) and 0.47 g (41%) of ethyl 4,5-dibromopentanoate (**4**). ^1^H NMR (400 MHz, CDCl_3_) δ: 1.25 (t, 3H, *J* = 7.1 Hz), 2.01 (m, 2H), 2.55 (m, 3H), 3.62 (dd, 1H, *J* = 10.4, 9.8), 3.85 (dd, 1H, *J* = 10.4, 4.5), 4.12 (q, 2H, *J* = 7.1), 4.23 (m, 1H).

**5**

**5-(Iodomethyl)-dihydrofuran-2(3*H*)-one** (**5**). A stirring bar, 0.22 mL of iodine monochloride, 3.0 mL of FC-72 and 0.40 mL of 4-pentenoic acid were placed in a 4 mL vial. The solution was stirred for 1 h. The product was dissolved in ethyl acetate and analyzed by GC-MS. Chromatography (Harrison Chromatotron, eluting with 3:1 hexanes/ethyl acetate) gave 0.78 g (86%) of 5-(iodomethyl)-dihydrofuran-2(3*H*)-one (**5**).

**8**

**Bromolactone** **8**. A stirring bar, 0.11 mL (2.2 mmol) of bromine, 1.0 mL of FC-72 and 276 mg (2 mmol) of 5-norbornylene-2-carboxylic acid (3:1 mixture of *endo* and *exo* isomers) were placed in a 4 mL vial. The solution was stirred for 1 h. The product was dissolved in ethyl acetate and analyzed by GC-MS. Chromatography (Harrison Chromatotron, eluting with 3:1 hexanes/ethyl acetate) gave 198 mg (48%) of bromolactone **8**.

**13**

**Iodolactone** **13**. A stirring bar, 0.12 mL (2.4 mmol) of iodine monochloride, 1.0 mL of FC-72 and 276 mg (2 mmol) of 5-norbornylene-2-carboxylic acid (5:1 mixture of *endo* and *exo* isomers) were placed in a 4 mL vial. The solution was stirred for 1 h. The product was dissolved in ethyl acetate and analyzed by GC-MS. Chromatography (Harrison Chromatotron, eluting with 3:1 hexanes/ethyl acetate) gave 343 mg (65%) of iodolactone **13**.

**15**

**Bromolactone acid** **15**. A stirring bar, 0.105 mL (2.1 mmol) of bromine, 1.0 mL of FC-72 and 364 mg (2 mmol) of 5-norbornylene-*endo*-2,3-dicarboxylic acid were placed in a 4 mL vial. The solution was stirred for 1 hour and the liquid was removed with a pipette. Bromolactone carboxylic acid **15** was not isolated. Instead, the solid product was dissolved in methanol (10 mL) and 0.5 mL of conc. sulfuric acid was added. The solution was refluxed for 1 h. The solution was partitioned between water and ethyl acetate and extracted with ethyl acetate (3 x 5 mL). The ethyl acetate extracts were rinsed with water, aqueous NaHCO_3_, aqueous NaCl, dried (anh. MgSO_4_), filtered and evaporated. Chromatography (Harrison Chromatotron, eluting with 3:1 hexanes/ethyl acetate) gave 396 mg (72%) of the methyl ester of bromolactone carboxylic acid **18**.

**16**

**Iodolactone acid 16** from a phase-vanishing reaction with iodine monochloride. A stirring bar, 0.115 mL (2.3 mmol) of iodine monochloride, 1.0 mL of FC-72 and 364 mg (2 mmol) of 5-norbornylene-*endo*-2,3-dicarboxylic acid were placed in a 4 mL vial. The solution was stirred for 1 hour and the liquid was removed with a pipette. Iodolactone carboxylic acid **16** was not isolated. Instead, the solid product was dissolved in methanol (10 mL) and 0.5 mL of conc. sulfuric acid was added. The solution was refluxed for 1 h. The solution was partitioned between water and ethyl acetate and extracted with ethyl acetate (3 x 5 mL). The ethyl acetate extracts were rinsed with water, aqueous NaHCO_3_, aqueous NaCl, dried (anh. MgSO_4_), filtered and evaporated. Chromatography (Harrison Chromatotron, eluting with 3:1 hexanes/ethyl acetate) gave 483 mg (75% total yield from 5-norbornylene-*endo*-2,3-dicarboxylic acid) of the methyl ester of iodolactone carboxylic acid **19**.

**Iodolactone acid 16** from a phase-vanishing reaction with iodine. A stirring bar, 508 mg (4 mmol) of finely ground iodine, 2.0 mL of FC-72 and 364 mg (2 mmol) of 5-norbornylene-*endo*-2,3-dicarboxylic acid were placed in a 4 mL vial. The solution was stirred for 3 days and the product was dissolved in ethyl acetate. The solution was extracted with aqueous sodium thiosulfate until it became colorless. Iodolactone carboxylic acid **16** was not isolated. Instead, the ethyl acetate extract was evaporated and the solid product was dissolved in methanol (10 mL) and 0.5 mL of conc. sulfuric acid. This solution was refluxed for 1 h. The solution was partitioned between water and ethyl acetate and extracted with ethyl acetate (3 x 5 mL). The ethyl acetate extracts were rinsed with water, aqueous NaHCO_3_, aqueous NaCl, dried (anh. MgSO_4_), filtered and evaporated. Chromatography (Harrison Chromatotron, eluting with 3:1 hexanes/ethyl acetate) gave 347 mg (54%) of methyl ester of iodolactone carboxylic acid **19**.

**18**

**Bromolactone ester** **18**. A stirring bar, 0.50 mL (10 mmol) of bromine, 3.0 mL of FC-72 and 2.10 g (10 mmol) of dimethyl 5-norbornylene-*endo*-2,3-dicarboxylate were placed in a 20 mL vial. The solution was stirred for 1 hour. The product was dissolved in ethyl acetate and chromatographed (2.5 cm x 12 cm silica gel column, eluting with 3:1 hexanes/ethyl acetate) to give 2.28 g (83%) of bromolactone ester **18**. ^1^H NMR (400 MHz, CDCl_3_) δ: ^1^H NMR (400 MHz, CDCl_3_) δ: 1.79 (br d, 1H, *J* = 11.7 Hz), 2.43 (br d, 1H, *J* = 11.7 Hz), 2.84 (dd, 1 H, *J* = 11.0, 4.7 Hz), 2.90 (m, 1H), 3.16 (dd, 1 H, *J* = 10.8, 3.4 Hz), 3.35 (m, 1 H), 3.74 (s, 3H), 4.61 (d, 1H, *J* = 2.4 Hz), 5.01 (d, 1H, *J* = 5.1 Hz).

**19**

**Iodolactone ester** **19**. A stirring bar, 0.90 mL (18 mmol) of iodine monochloride, 4.0 mL of FC-72 and 3.15 g (15 mmol) of dimethyl 5-norbornylene-*endo*-2,3-dicarboxylate were placed in a 20 mL vial. The solution was stirred for 1 hour. The product was dissolved in ethyl acetate, treated with aqueous sodium thiosulfate and chromatographed (2.5 cm x 12 cm silica gel column, eluting with 3:1 hexanes/ethyl acetate) to give 4.15 g (86%) of iodolactone ester **19**.

**21**

**Bromolactone ester** **21**. A stirring bar, 0.50 mL (10 mmol) of bromine, 3.0 mL of FC-72 and 2.10 g (10 mmol) of dimethyl 5-norbornene-2-*endo*,3-*exo*-dicarboxylate were placed in a 20 mL vial. The solution was stirred for 30 min. The product was dissolved in ethyl acetate and filtered through a short column of silica gel (2.5 cm x 3 cm, eluting with 1:1 hexanes/ethyl acetate) to give 2.53 g (92%) of bromolactone ester **21**. ^1^H NMR (400 MHz, CDCl_3_) δ: 1.89 (m, 1H), 2.28 (br d, 1H, *J* = 11.9 Hz), 2.79 (m, 1 H), 2.99 (m, 1H), 3.11 (m, 1H), 3.26 (m, 1 H), 3.75 (s, 3H), 3.85 (m, 1H), 4.94 (d, 1H, *J* = 5.1 Hz).

**22**

**Iodolactone ester** **22**. A stirring bar, 1.50 mL (30 mmol) of iodine monochloride, 5.0 mL of FC-72 and 5.25 g (25 mmol) of dimethyl 5-norbornene-2-*endo*,3-*exo*-dicarboxylate were placed in a 20 mL vial. Solid starting material formed a melt and then solidified again. The solution was stirred for 1 hour. The product was dissolved in ethyl acetate, treated with aqueous sodium thiosulfate and filtered through a short column of silica gel (2.5 cm x 3 cm, eluting with 1:1 hexanes/ethyl acetate) to give 7.57 g (94%) of iodolactone ester **22**.

**References**

1. Abe, M.; You, Y.; Detty, M. R. *Organometallics* **2002,** *21,* 4546–4551. doi:[10.1021/om020511p](http://dx.doi.org/10.1021/om020511p)
2. Curran, D. P.; Tamine, J. *J. Org. Chem.* **1991,** *56,* 2746–2750. doi:[10.1021/jo00008a032](http://dx.doi.org/10.1021/jo00008a032)
3. Mellegaard, S. R.; Tunge, J. A. *J. Org. Chem.* **2004,** *69,* 8979–8981. doi:[10.1021/jo048460o](http://dx.doi.org/10.1021/jo048460o)
4. Moriarty, R. M.; Gopal, H.; Walsh, H. G.; Ramey, K. C.; Lini, D. C. *Tetrahedron Lett.* **1966,** 4555–4560. doi:[10.1016/S0040-4039(00)70077-3](http://dx.doi.org/10.1016/S0040-4039(00)70077-3)
5. Zefirov, N. S.; Sereda, G. A.; Sosonuk, S. E.; Zyk, N. V. Likhomanova, T. I. *Synthesis* **1995,** 1359–1361. doi:[10.1055/s-1995-4105](http://dx.doi.org/10.1055/s-1995-4105)
6. Kishikawa, K.; Horie, K.; Yamamoto, M.; Kohmoto, S.; Yamada, K. *Chem. Lett.* **1990,** 1009–1010. doi:[10.1246/cl.1990.1009](http://dx.doi.org/10.1246/cl.1990.1009)
7. Evans, R. M.; Owen, L. N. *J. Chem. Soc.* **1949,** 244–248. doi:[10.1039/jr9490000244](http://dx.doi.org/10.1039/jr9490000244)
8. Fleming, I.; Michael, J. P. *J. Chem. Soc., Perkin Trans. 1* **1981,** 1549–1556. doi:[10.1039/P19810001549](http://dx.doi.org/10.1039/P19810001549)
