# Supplementary material for: Phase-vanishing halolactonization of neat substrates
Source: Beilstein J Org Chem. 2008 Aug 11;4:29. doi: 10.3762/bjoc.4.29 (PMC2533434; doi:10.3762/bjoc.4.29)
Supplement: File 2 — GC-MS and NMR spectra [file Beilstein_J_Org_Chem-04-29-s002.pdf]

Supporting Information

Phase-vanishing halolactonization of neat substrates

Nicole Windmon[1] and Veljko Dragojlovic[2,*]

[1]Department of Chemistry, Florida Atlantic University, 777 Glades Road, Boca Raton, Florida 33431, USA.

[2]Wilkes Honors College of Florida Atlantic University, 5353 Parkside Drive, Jupiter, Florida, 33458, USA.

Veljko Dragojlovic – vdragojl@fau.edu

*corresponding author

GC-MS spectra of the compounds **2**, **3**, **4**, **5**, **8**, **13**, **18**, **19**, **21**, **22** and ^{1}H NMR spectra of the compounds **2**, **4**, **5**, **8**, **13**, **18**, **19**, **21**, **22**.

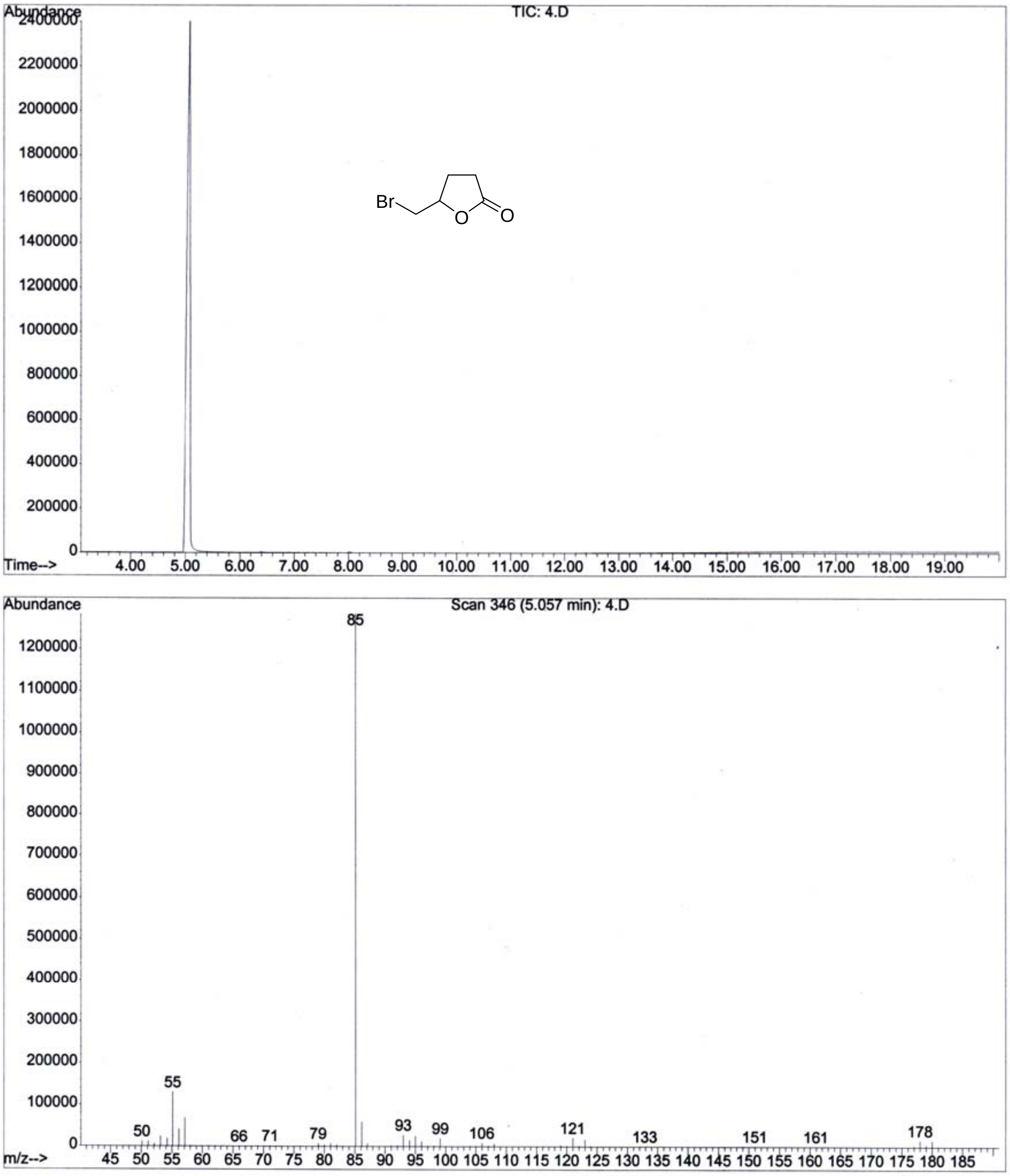

GC-MS of 5-(bromomethyl)-dihydrofuran-2(3*H*)-one (**2**).

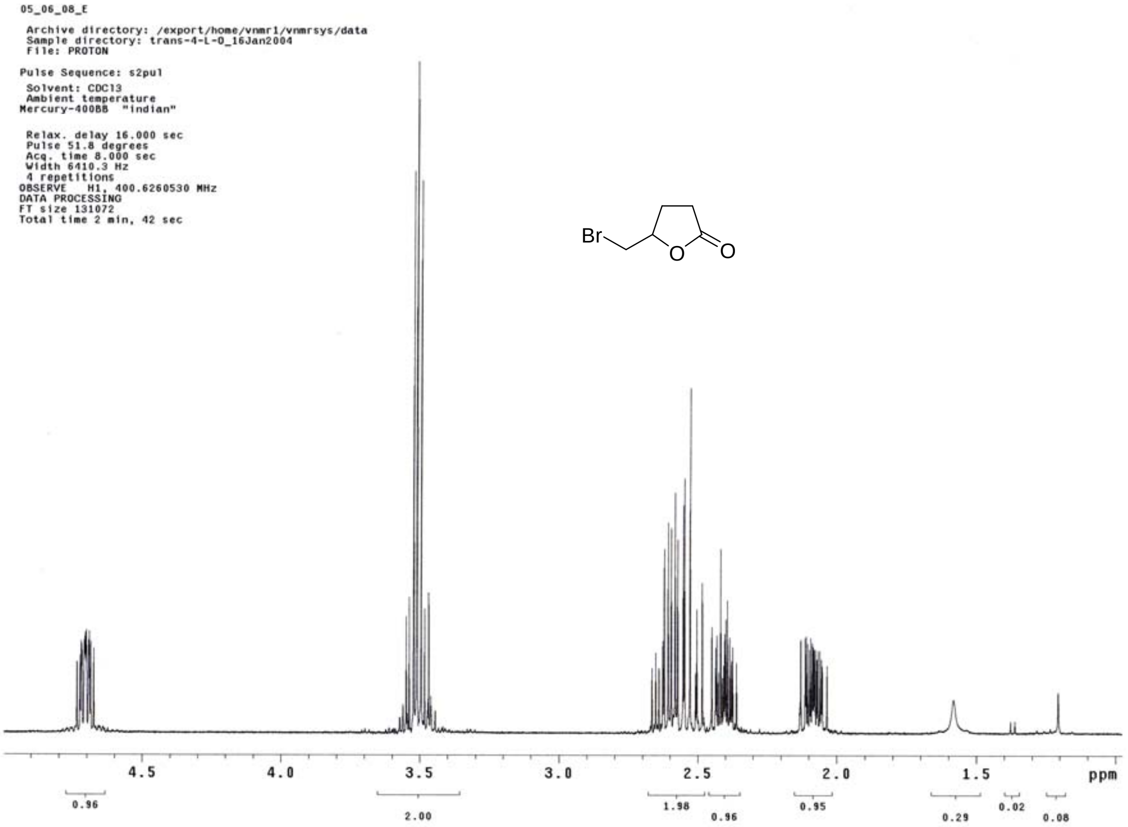

^{1}H NMR of 5-(bromomethyl)-dihydrofuran-2(3*H*)-one (**2**).

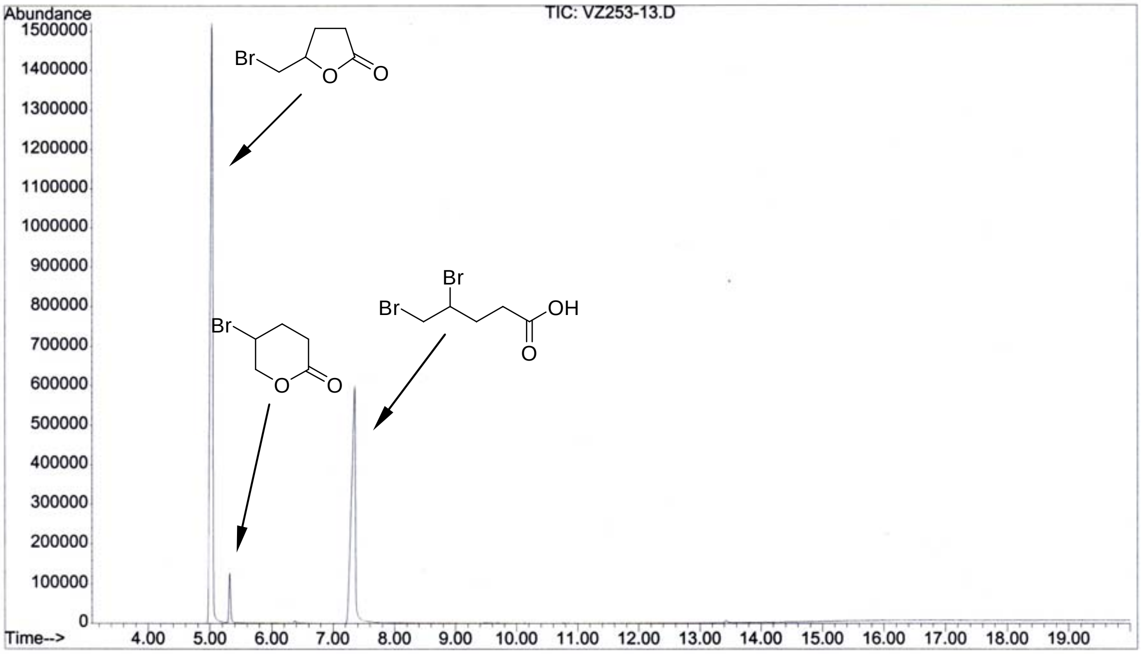

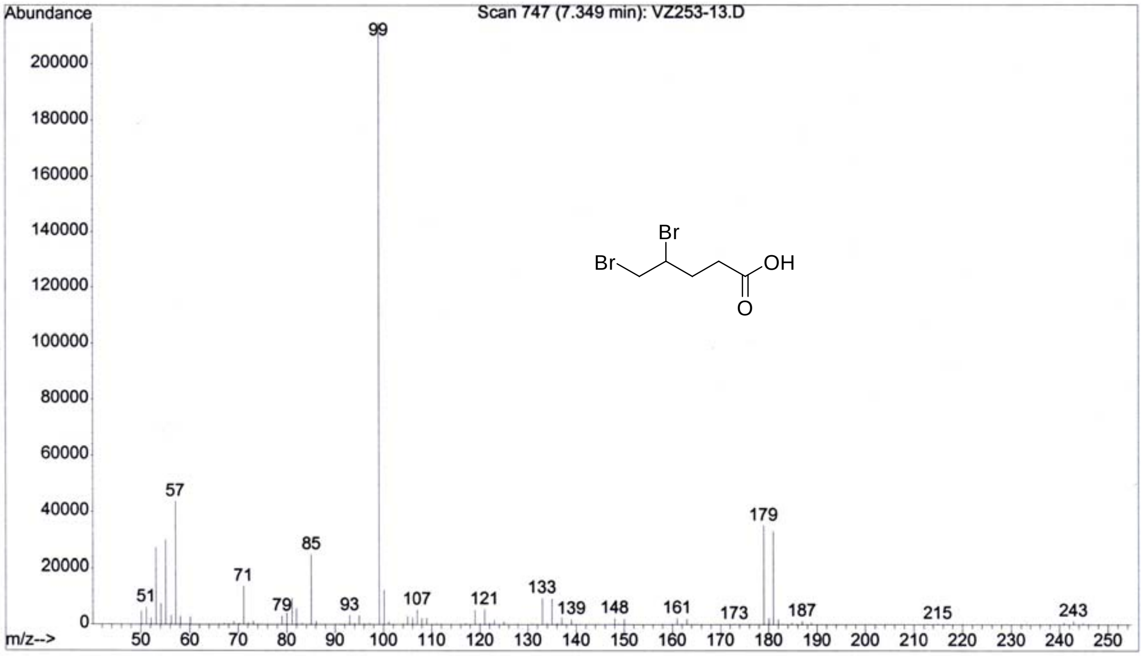

GC-MS of solvent-free reaction of bromine with 4-pentenoic acid. The reaction mixture contains 4,5-dibromopentanoic acid (**3**).

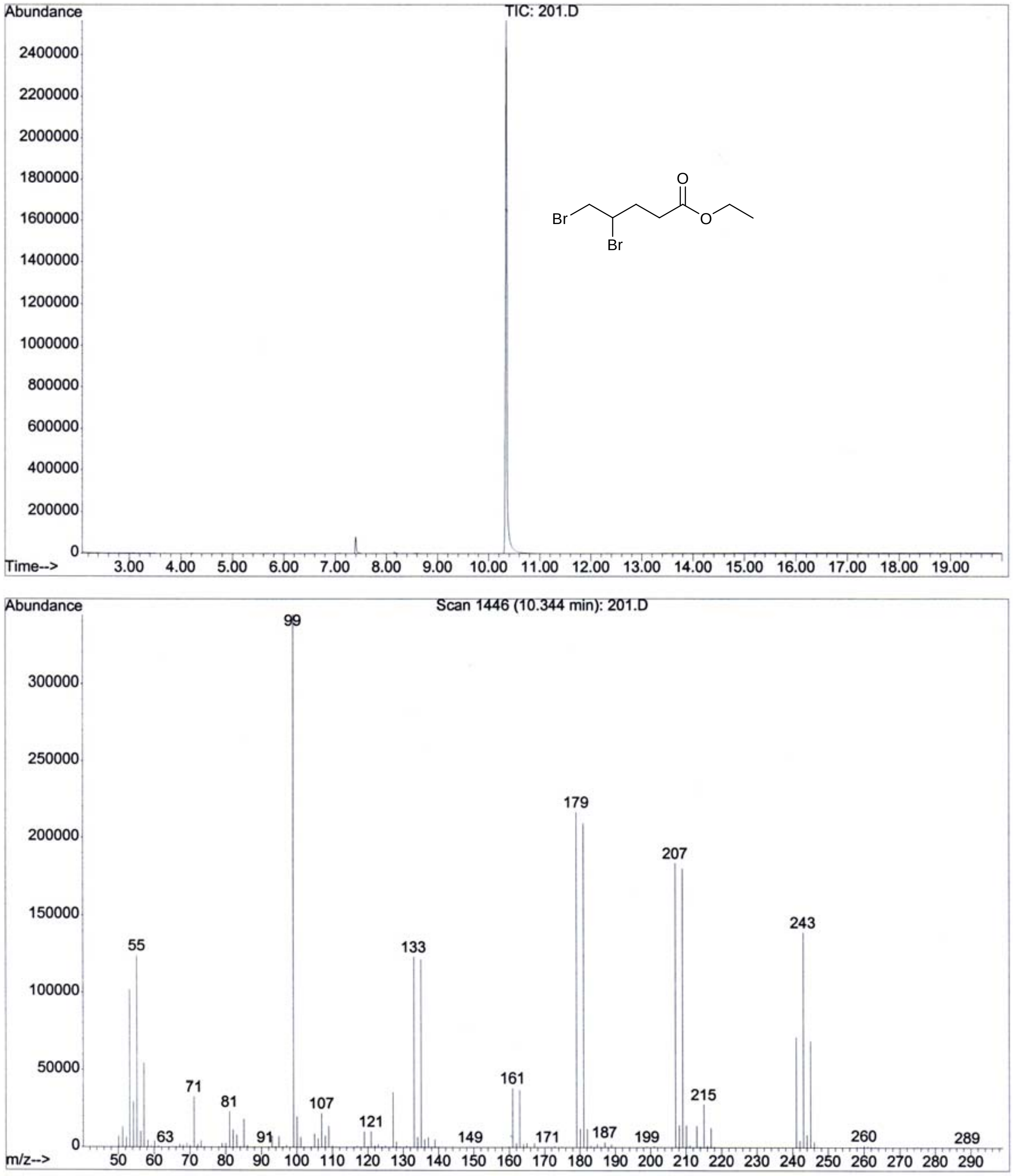

GC-MS of ethyl 4,5-dibromopentanoate (**4**).

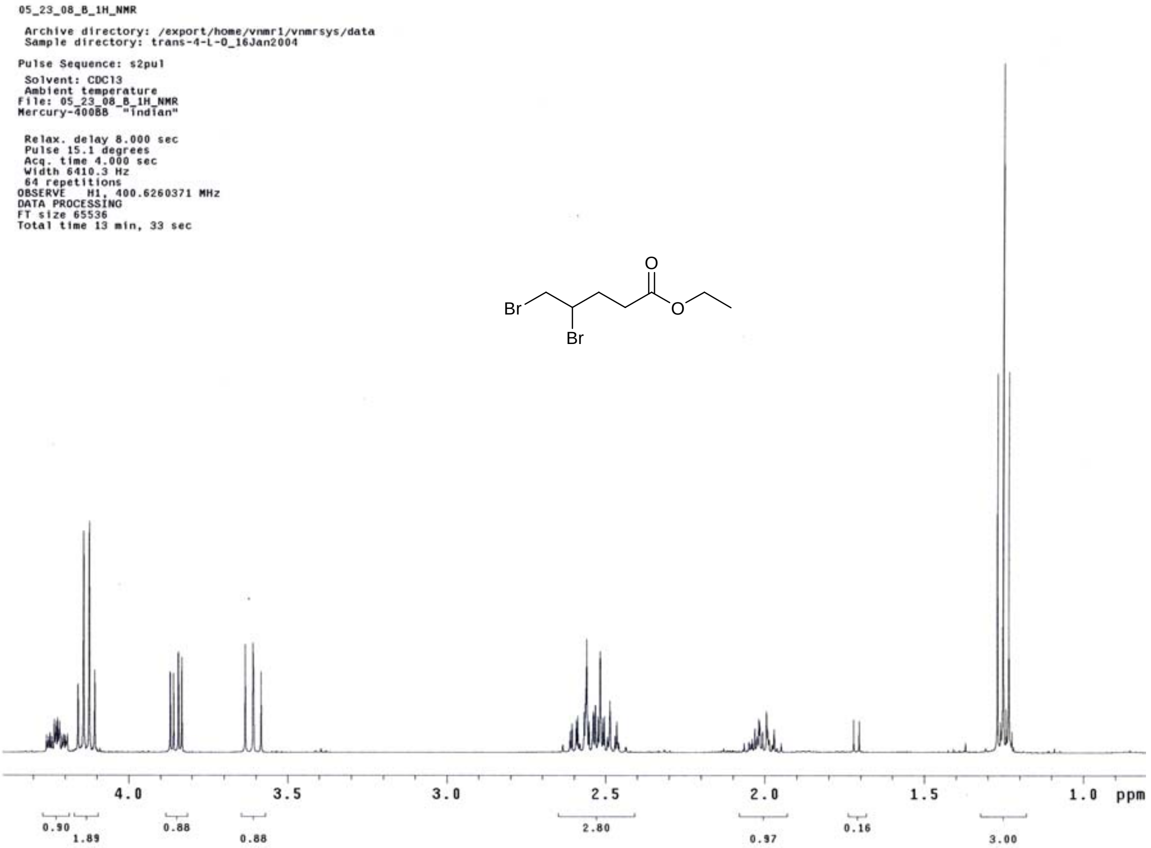

^{1}H NMR of ethyl 4,5-dibromopentanoate (**4**).

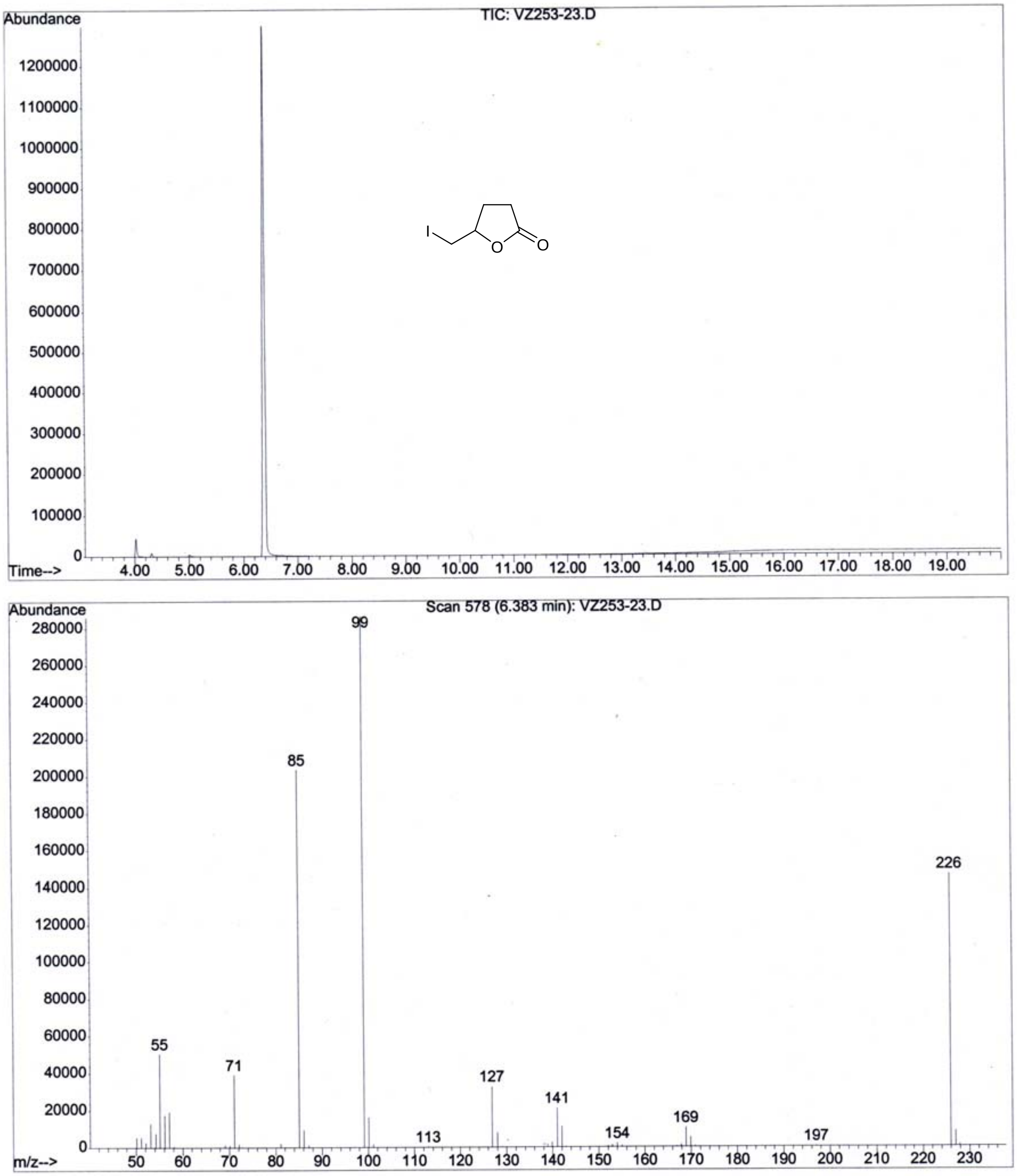

GC-MS of 5-(iodomethyl)-dihydrofuran-2(3*H*)-one (**5**).

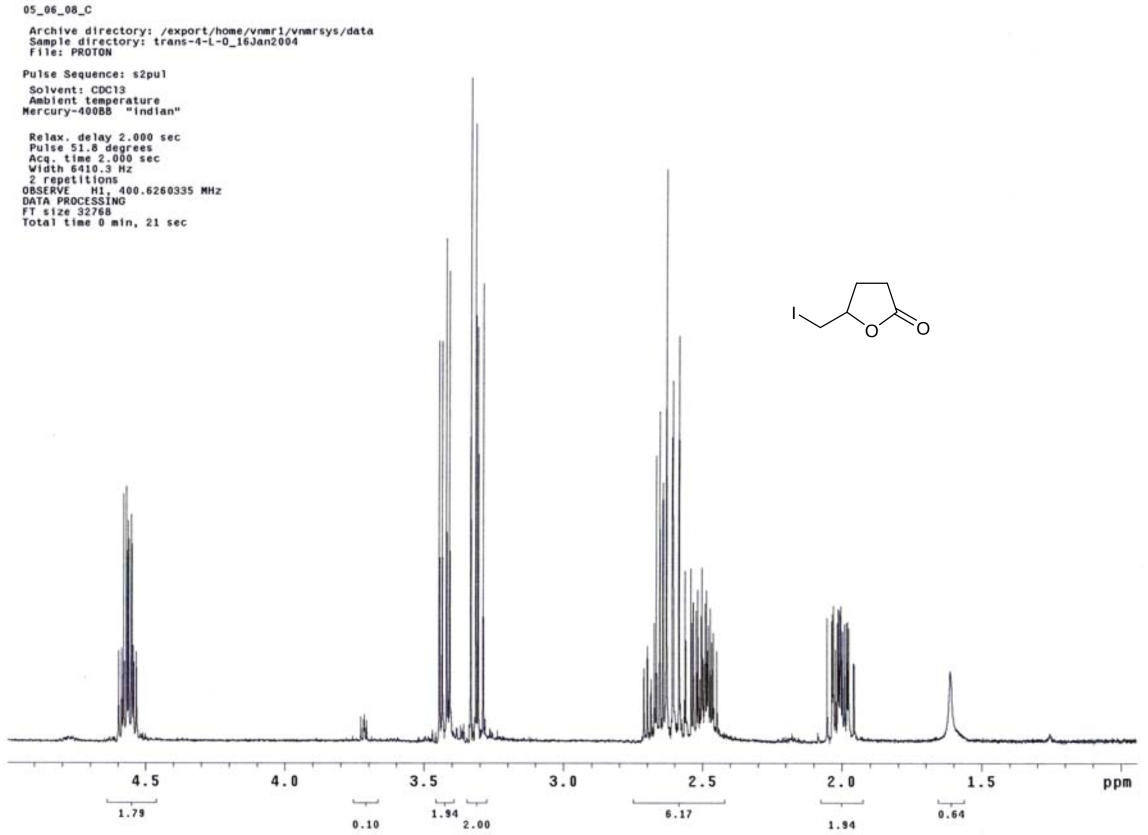

^{1}H NMR of 5-(iodomethyl)-dihydrofuran-2(3*H*)-one (**5**).

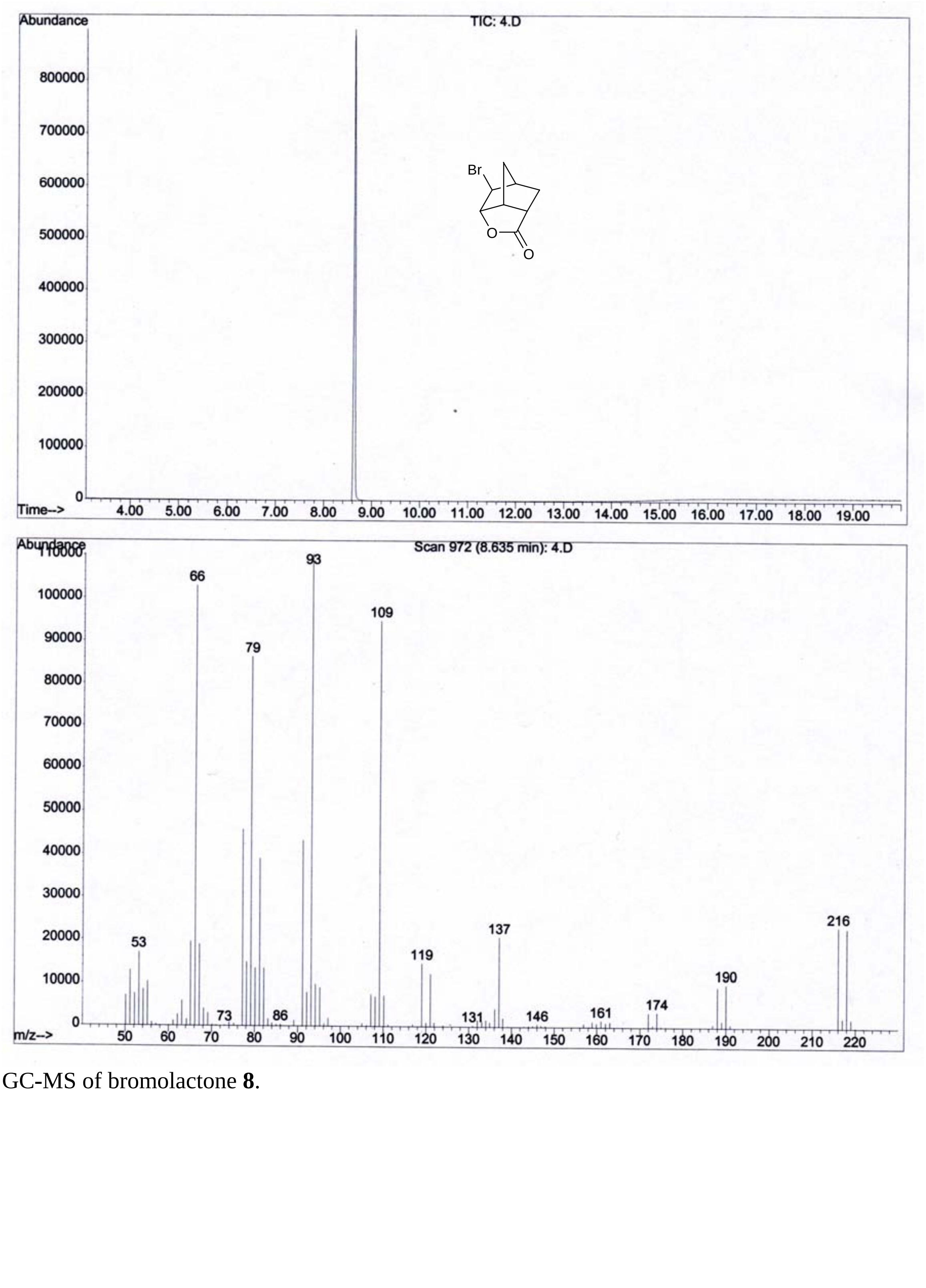

GC-MS of bromolactone **8**.

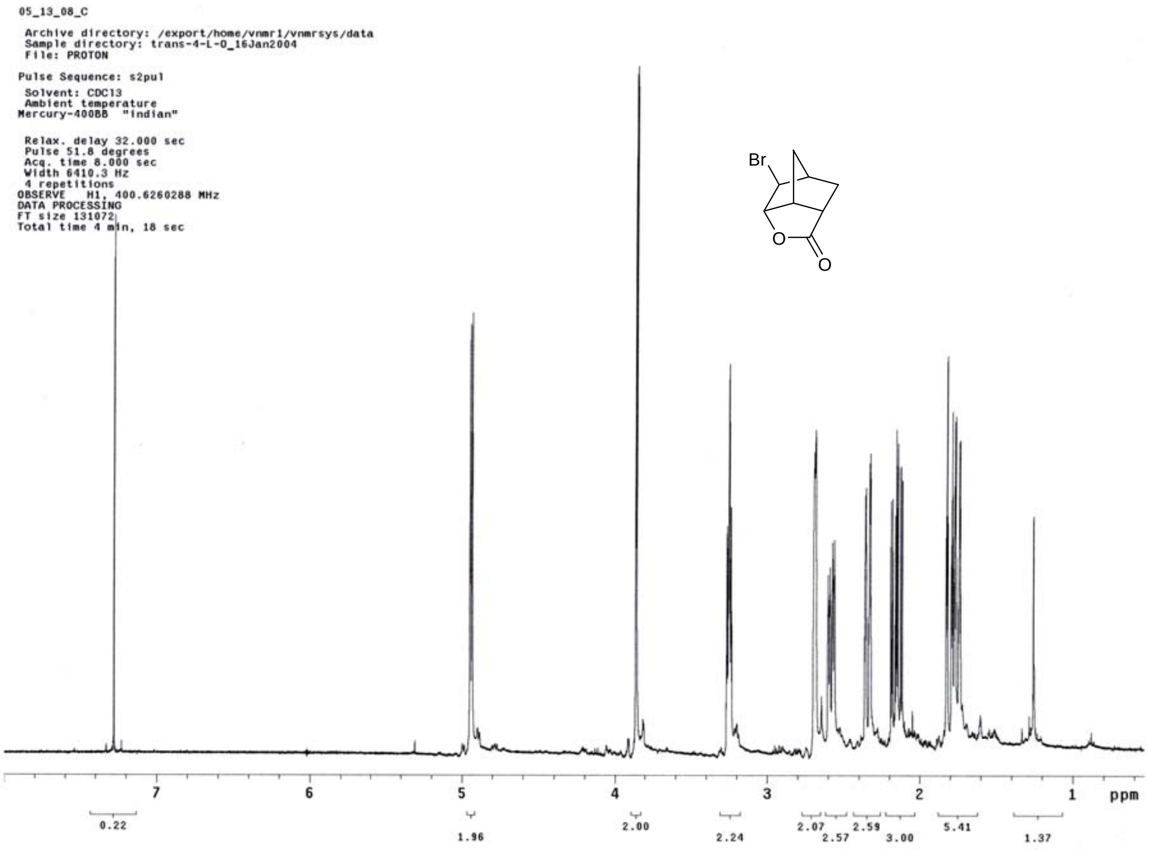

^{1}H NMR of bromolactone **8**.

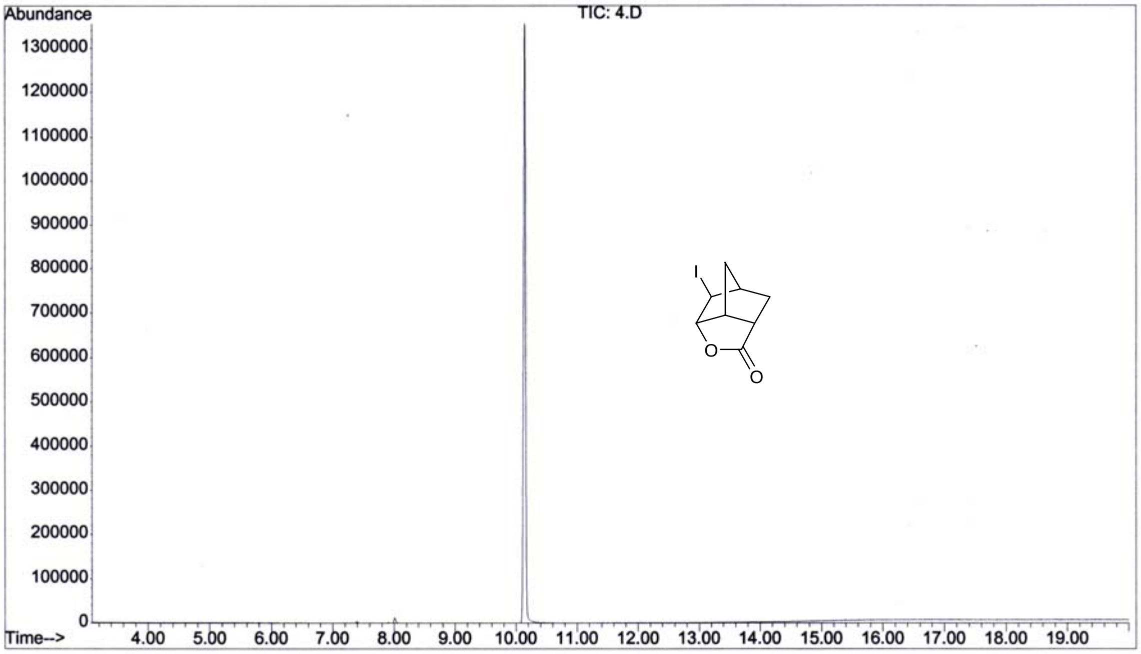

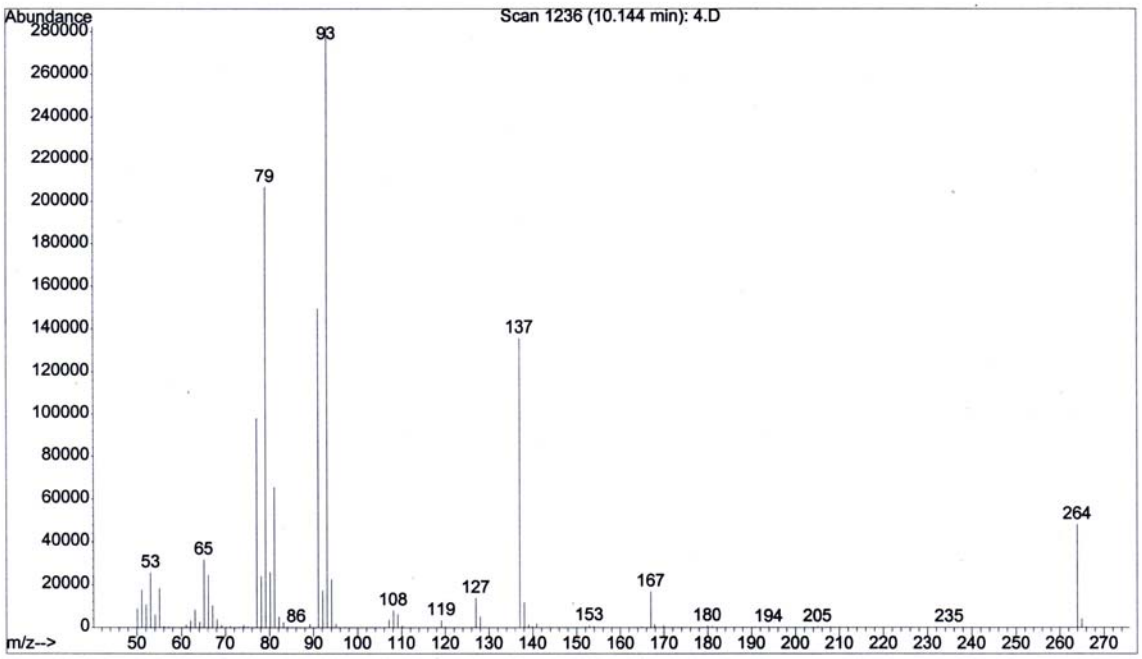

GC-MS of iodolactone **13**.

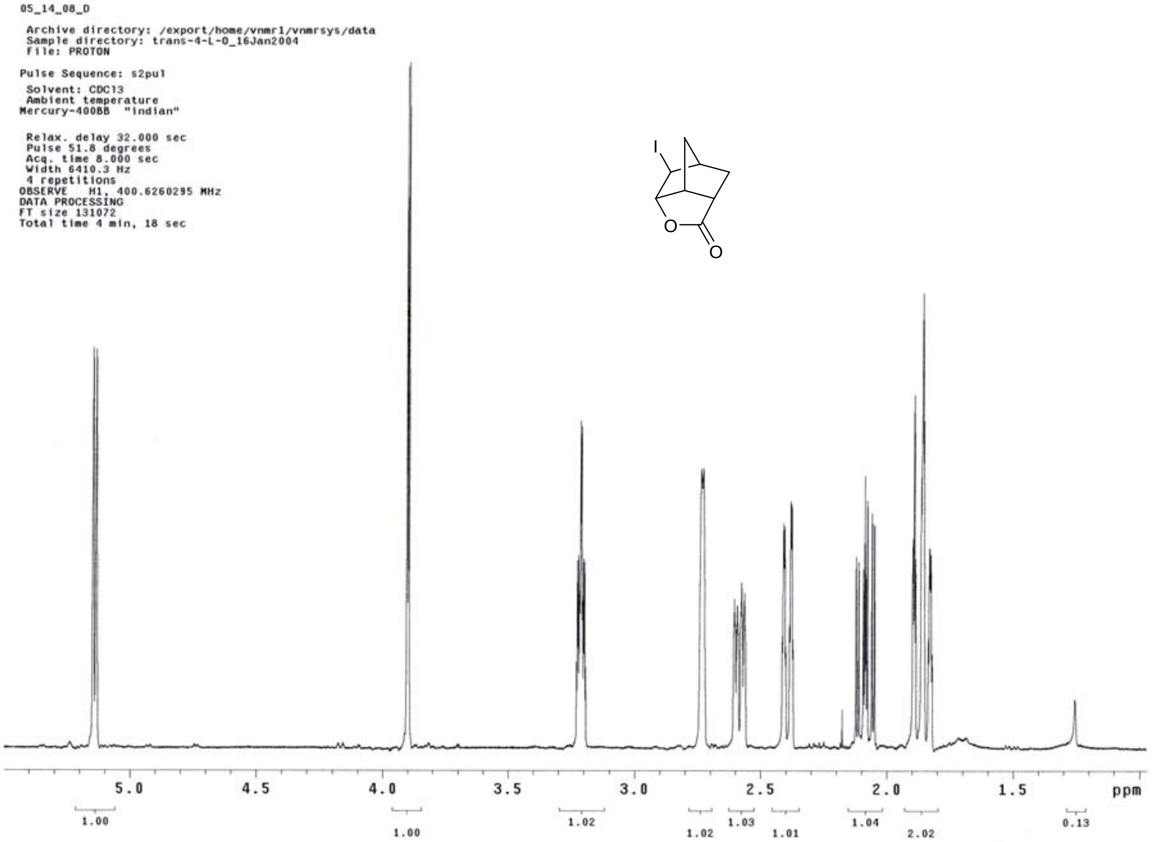

^{1}H NMR of iodolactone **13**.

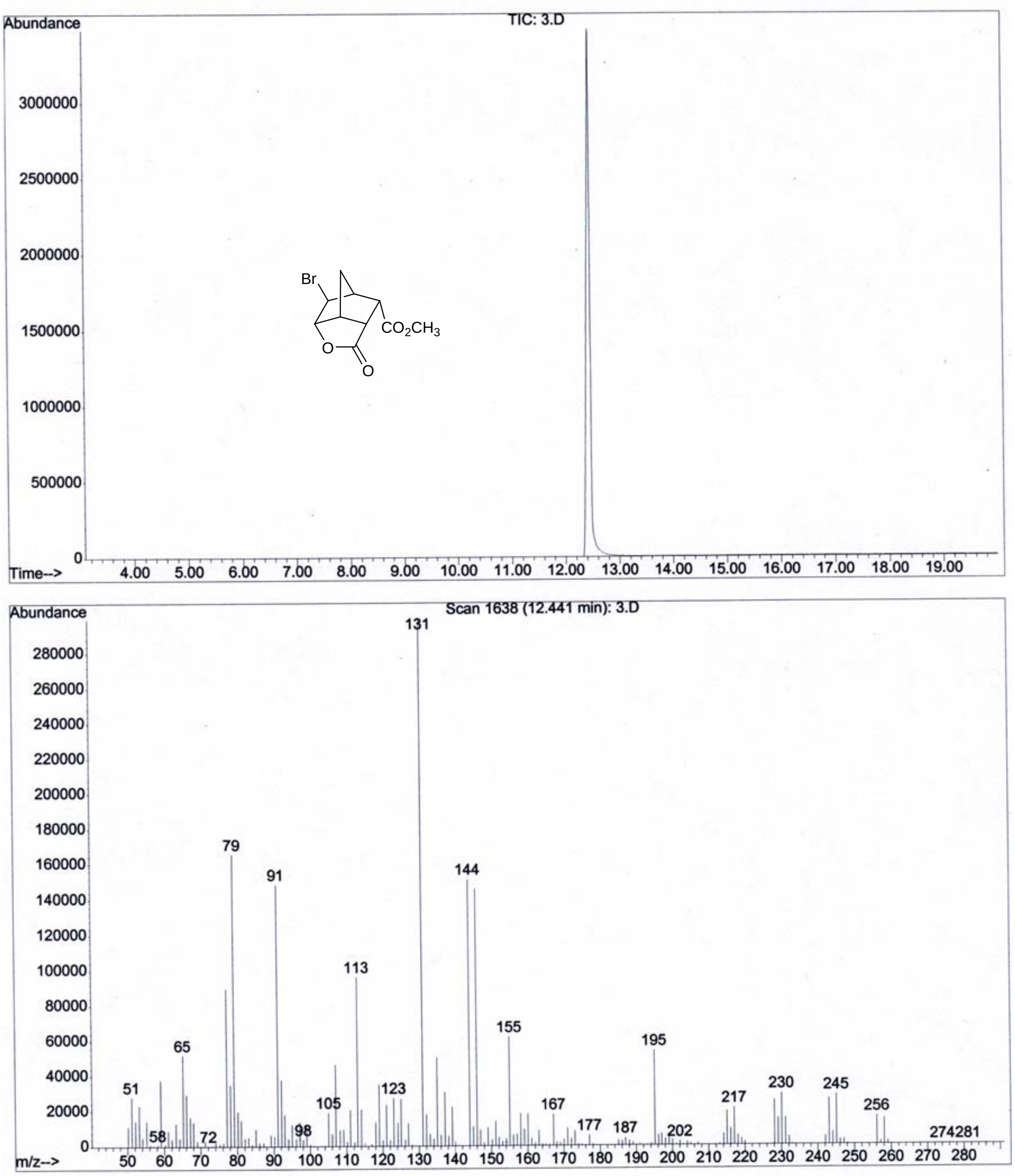

GC-MS of bromolactone ester **18**.

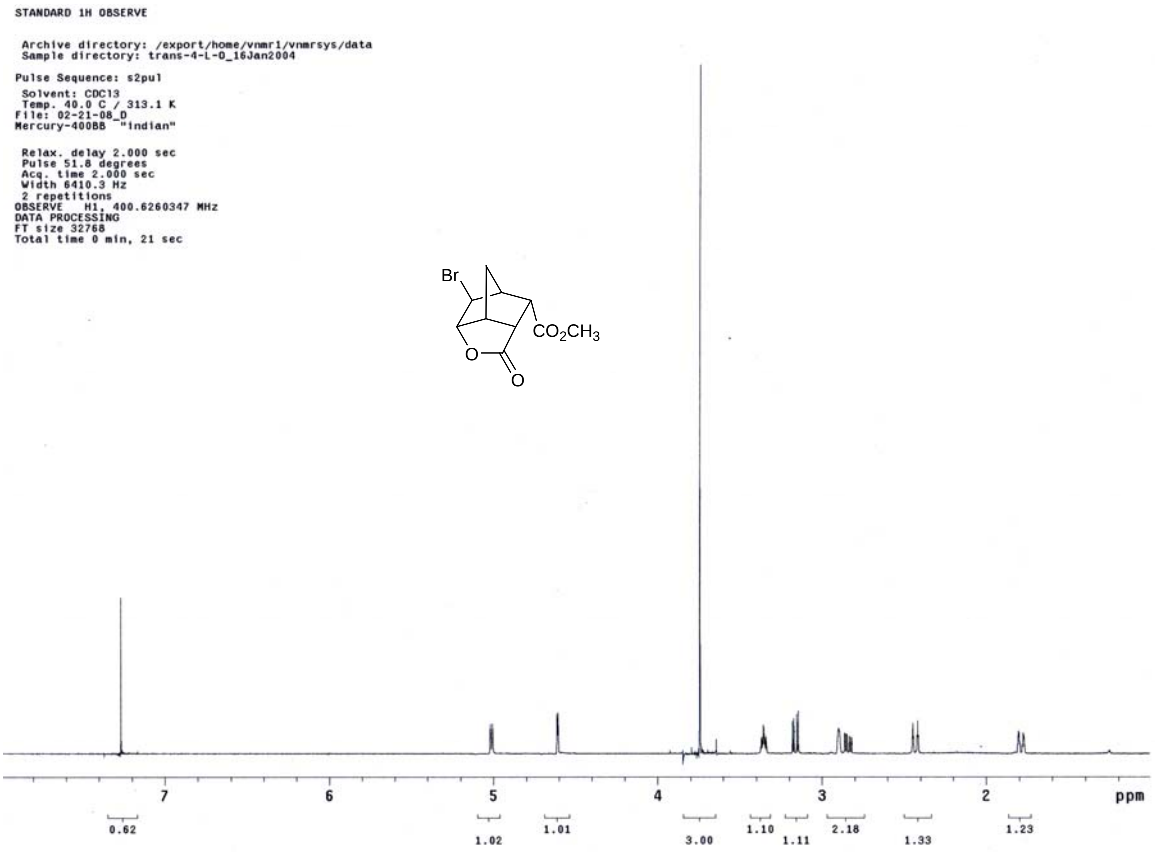

^{1}H NMR of bromolactone ester **18**.

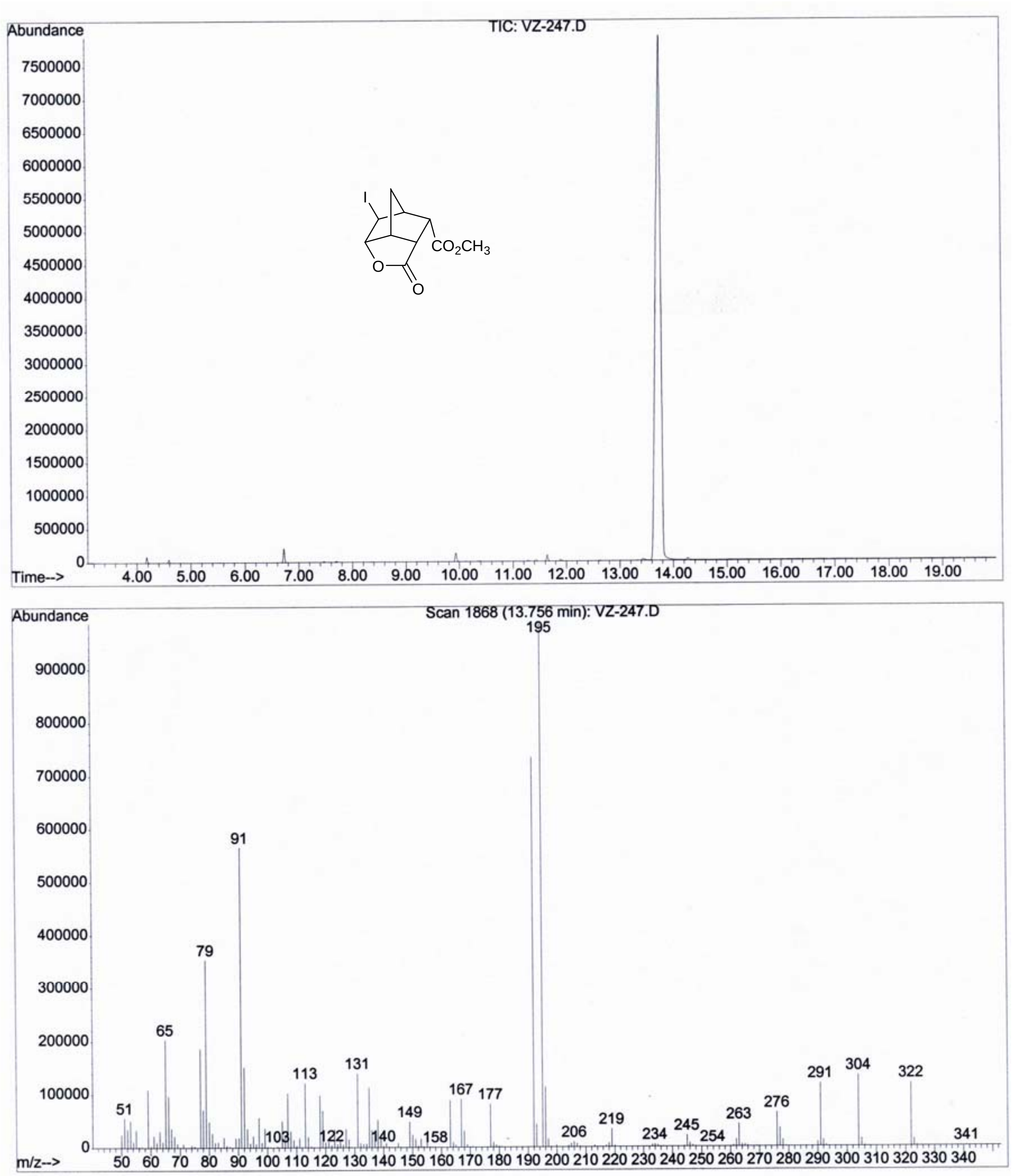

GC-MS of iodolactone ester **19**.

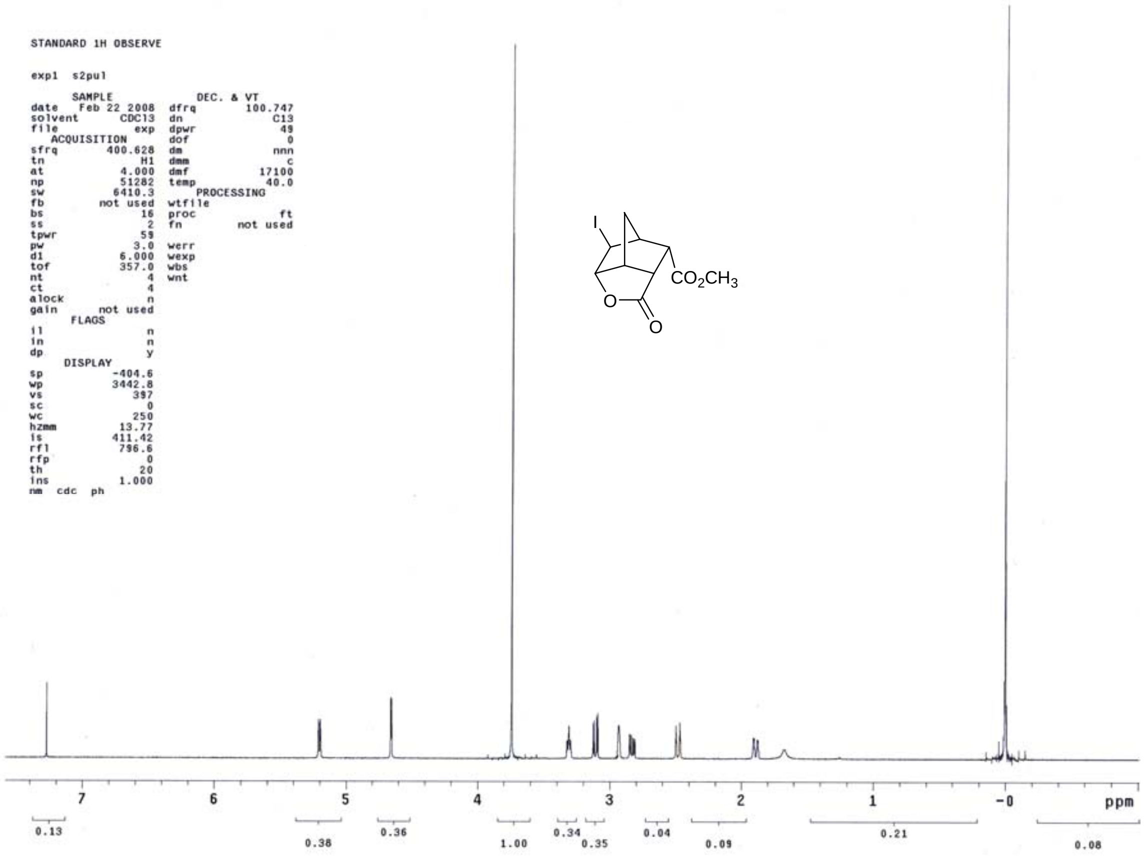

^{1}H NMR of iodolactone ester **19**.

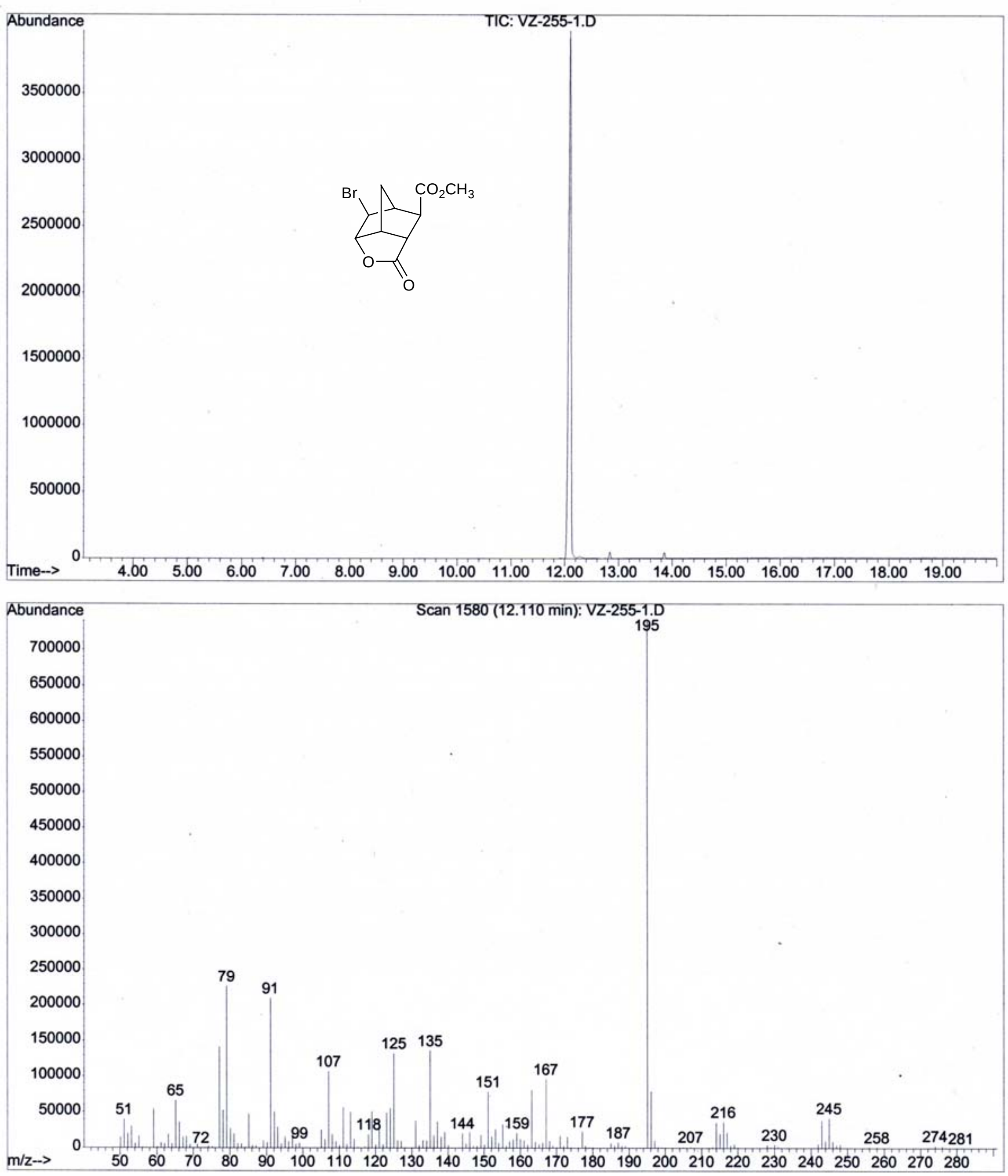

GC-MS of bromolactone ester **21**.

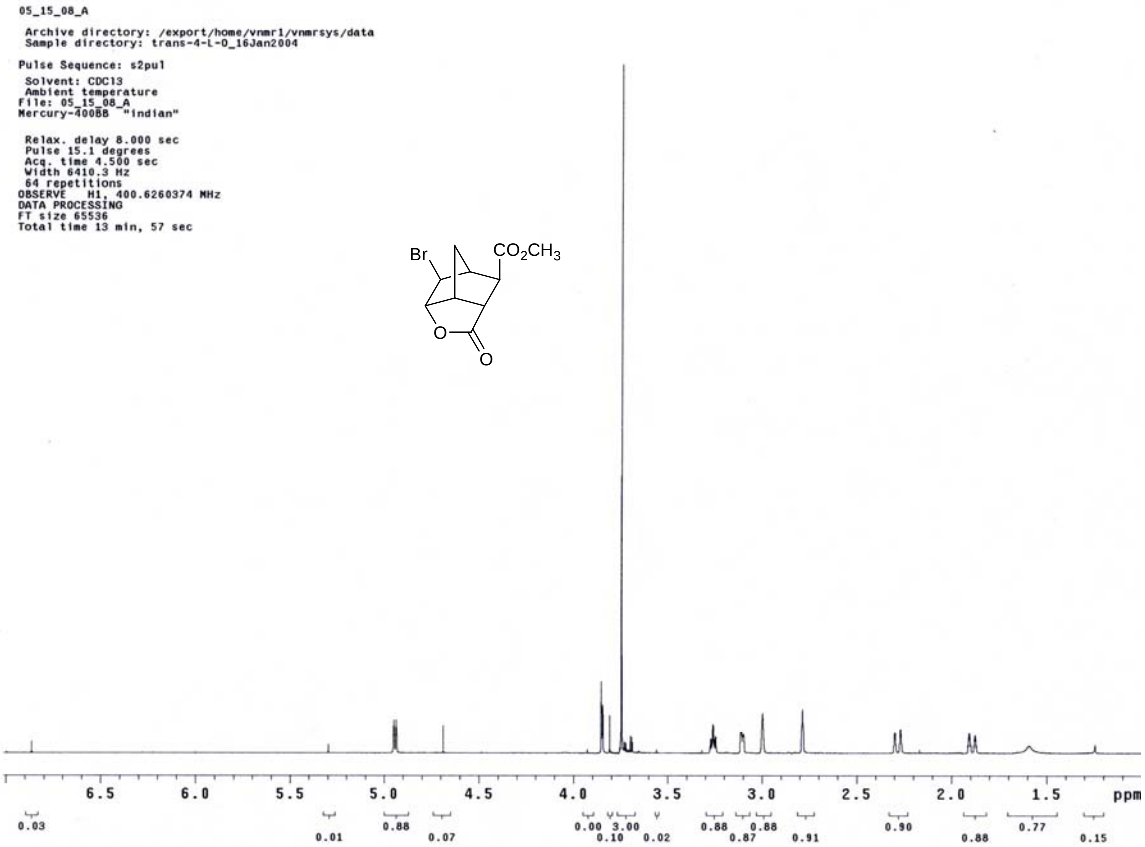

^{1}H NMR of bromolactone ester **21**.

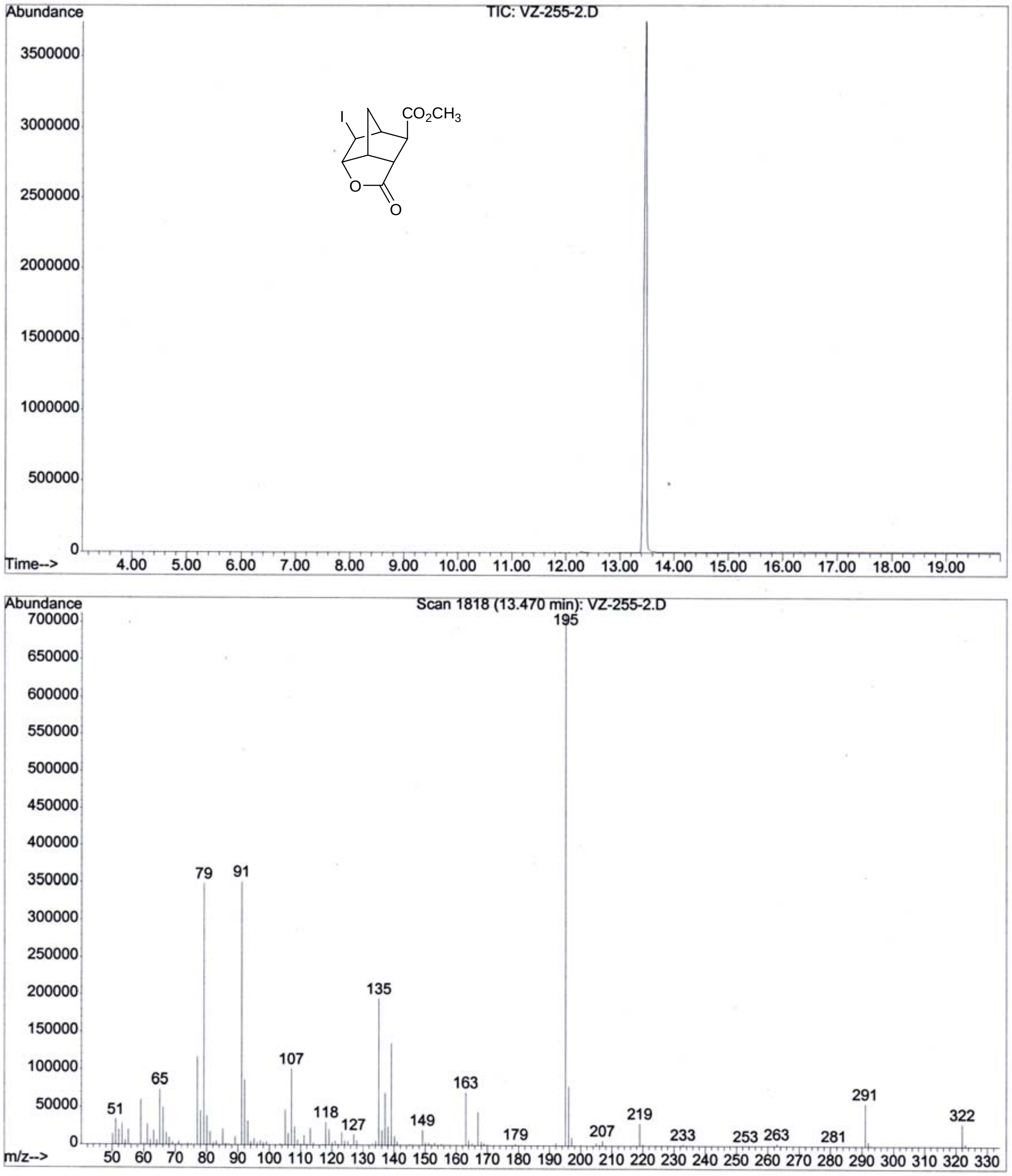

GC-MS of iodolactone ester **22**.

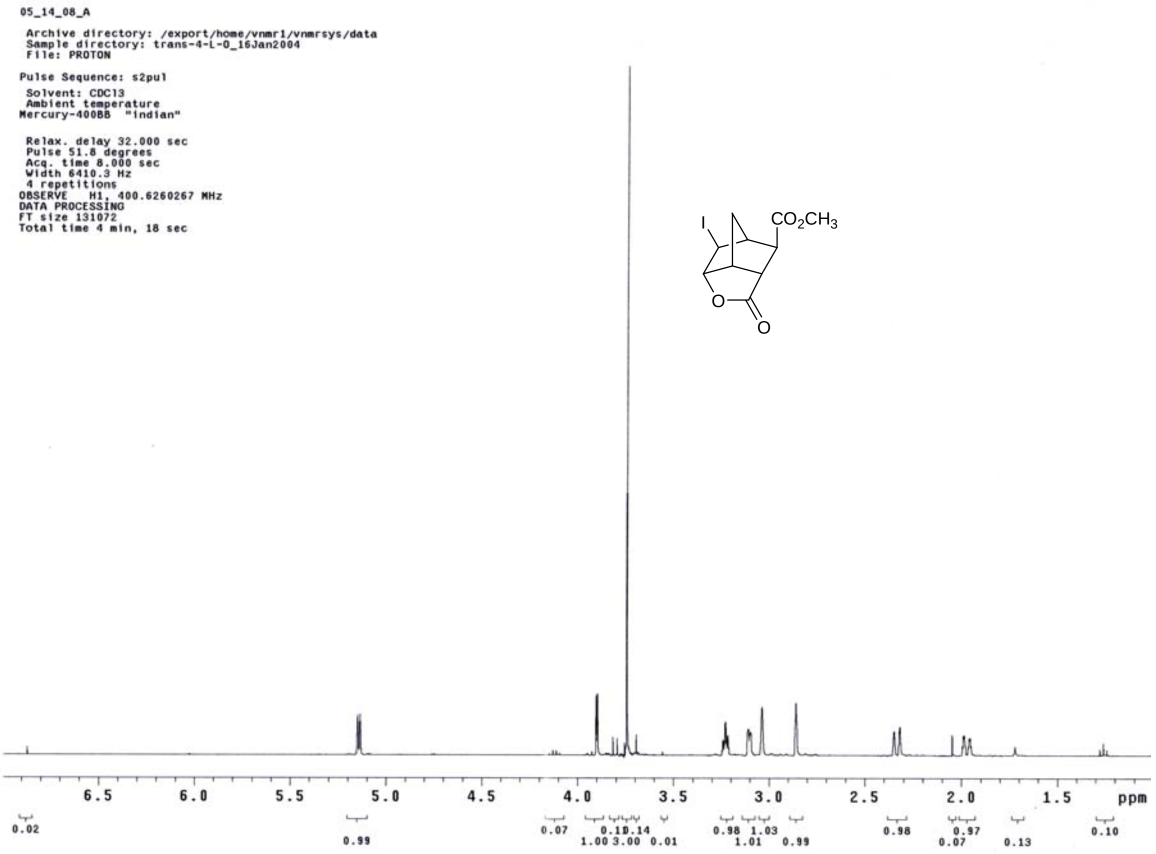

^{1}H NMR of iodolactone ester **22**.
